# Effects of changes in trunk inclination on ventilatory efficiency in ARDS patients: quasi-experimental study

**DOI:** 10.1186/s40635-023-00550-2

**Published:** 2023-09-27

**Authors:** Martín H. Benites, David Torres, Fabian Poblete, Francisco Labbe, María C. Bachmann, Tomas E. Regueira, Leonardo Soto, Andrés Ferre, Jorge Dreyse, Jaime Retamal

**Affiliations:** 1 Unidad de Pacientes Críticos, Clínica Las Condes, Estoril 450, Santiago, Chile; 2 Departamento de Epidemiología y Estudios en Salud, Magíster en Epidemiología, Universidad de los Andes, Monseñor Álvaro del Portillo 12455, Santiago, Chile; 3 Estudiante del Programa Doctorado en Ciencias Médicas, Escuela de Medicina, Pontificia Universidad Católica de Chile, Av. Libertador Bernardo O’Higgins 340, Santiago, Chile; 4 Facultad de Medicina, Escuela de Medicina, Universidad Finis Terrae, Av. Pedro de Valdivia 1509, Santiago, Chile; 5 Departamento de Medicina Intensiva, Hospital Clínico Pontificia Universidad Católica de Chile, Marcoleta 367, Santiago, Chile; 6 Unidad de Pacientes Críticos, Clínica Santa María, Bellavista 415, Santiago, Chile

**Keywords:** Acute respiratory distress syndrome, Body position, Respiratory dead space, Tidal volume

## Abstract

**Background:**

Trunk inclination from semirecumbent head-upright to supine-flat positioning reduces driving pressure and increases respiratory system compliance in patients with acute respiratory distress syndrome (ARDS). These effects are associated with an improved ventilatory ratio and reduction in the partial pressure of carbon dioxide (PaCO_2_). However, these physiological effects have not been completely studied, and their mechanisms have not yet been elucidated. Therefore, this study aimed to evaluate the effects of a change in trunk inclination from semirecumbent (45°) to supine-flat (10°) on physiological dead space and ventilation distribution in different lung regions.

**Results:**

Twenty-two ARDS patients on pressure-controlled ventilation underwent three 60-min steps in which trunk inclination was changed from 45° (baseline) to 10° (intervention) and back to 45° (control) in the last step. Tunk inclination from a semirecumbent (45°) to a supine-flat (10°) position resulted in a higher tidal volume [371 (± 76) vs. 433 (± 84) mL (*P* < 0.001)] and respiratory system compliance [34 (± 10) to 41 (± 12) mL/cmH_2_O (*P* < 0.001)]. The CO_2_ exhaled per minute improved from 191 mL/min (± 34) to 227 mL/min (± 38) (*P* < 0.001). Accordingly, Bohr’s dead space ratio decreased from 0.49 (± 0.07) to 0.41 (± 0.06) (*p* < 0.001), and PaCO_2_ decreased from 43 (± 5) to 36 (± 4) mmHg (*p* < 0.001). In addition, the impedance ratio, which divides the ventilation activity of the ventral region by the dorsal region ventilation activity in tidal images, dropped from 1.27 (0.83–1.78) to 0.86 (0.51–1.33) (*p* < 0.001). These results, calculated from functional EIT images, indicated further ventilation activity in the dorsal lung regions. These effects rapidly reversed once the patient was repositioned at 45°.

**Conclusions:**

A change in trunk inclination from a semirecumbent (45 degrees) to a supine-flat position (10 degrees) improved Bohr’s dead space ratio and reduced PaCO_2_ in patients with ARDS. This effect is associated with an increase in tidal volume and respiratory system compliance, along with further favourable impedance ventilation distribution toward the dorsal lung regions. This study highlights the importance of considering trunk inclination as a modifiable determinant of physiological parameters. The angle of trunk inclination is essential information that must be reported in ARDS patients.

**Supplementary Information:**

The online version contains supplementary material available at 10.1186/s40635-023-00550-2.

## Background

Changes in trunk inclination from a semirecumbent head-up position to a supine-flat position or vice versa can generate physiological effects that are relevant to daily clinical practice [1, 2]. These effects are related to respiratory mechanics, oxygenation, end-expiratory lung volume, partial pressure of carbon dioxide (PaCO_2_), and ventilatory ratio (VR) in patients with acute respiratory distress syndrome (ARDS) [1–3].

Several studies have found that semirecumbent head-upright positioning results in impaired respiratory system mechanics compared with supine-flat positions [1–6]. Moreover, although oxygenation can be improved in some patients, it is likely to occur in those who show increased end-expiratory lung volume (EELV) in a seated position [2]. The mechanism underlying these findings is not completely clear. However, semirecumbent positioning has been proposed to increase chest wall elastance and driving pressures, operating in a more ascendant part of the pressure‒volume curves, thereby inducing a caudal shift of the diaphragm and abdominal content. This shift likely minimizes atelectasis formation and enhances oxygenation in certain patients. [6, 7]. However, this increase in lung mechanical stress could generate alveolar strain in previously open regions.

Conversely, evidence suggests that changes in trunk inclination from a semirecumbent to a supine-flat position can decrease PaCO_2_ and VR [8, 9]. Nevertheless, the impact of changes in trunk inclination on alveolar ventilation and dead space still needs to be understood more, given that neither PaCO_2_ nor VR accurately measures ventilatory efficiency or inefficiency. This is because both variables reflect a combination of mechanisms influencing the ventilation/perfusion ratio (V̇/Q ˙) [9, 10]. Therefore, the effects of changes in body position on ventilatory efficiency/inefficiency in ventilated ARDS patients must be elucidated with a methodology that allows for more accurate measurement of alveolar ventilation and dead space. Hence, the primary objective of this study was to assess the effects of trunk inclination changes from 45° to 10° on PaCO_2_ levels and Bohr's dead space ratio (VD_Bohr_/V_T_). We hypothesized that changes in trunk inclination would improve CO_2_ removal efficiency. The secondary aim was to evaluate the distribution of ventilation in the different lung regions.

## Methods

A quasi-experimental, single-group study with repeated measures was performed in the medical ICU of Clínica las Condes, Santiago, Chile. The study was conducted between June 2021 and July 2022 (NCT05281536 ClinicalTrials.gov). The protocol was approved by the appropriate Institutional Review Board (Ethics Committee, Protocol number: E012021, Clínica las Condes approval date: January 14, 2021). Written informed consent was obtained from the patient’s next of kin. The procedures were conducted in accordance with the ethical standards of the Institutional Committee on Human Experimentation and the Helsinki Declaration of 1975.

### Patients

The inclusion and exclusion criteria were as follows:

Inclusion criteria:

1. ARDS patients.
2. Passive breathing through neuromuscular blockers or deep sedation to suppress all evidence of respiratory muscle activation.

Exclusion criteria:

1. Patients with gastric contents greater than 300 ml.
2. Patients with hemodynamic instability, as defined in a previous study [11].
3. Patients with variations in esophageal temperature exceeding 0.5 °C within the last 2 h [12].
4. Contraindications to the placement of electrical impedance tomography (EIT) belts (implantable cardiac pacemaker or defibrillator and wounds that limited electrode belt placement).

Furthermore, enteral nutrition was suspended immediately before starting the study to minimize the potential risk of gastric content regurgitation.

### Data collection

Age, sex, height, body mass index, Acute Physiology and Chronic Health Evaluation II score, number of days of mechanical ventilation, and ARDS etiology were recorded at baseline.

### Baseline ventilatory strategy

Mechanical ventilation at baseline was programmed using a pressure‒controlled ventilation mode (Dräger Evita Infinity^®^ V500, Germany) with the following parameters: peak inspiratory pressure set to reach a tidal volume (V_T_) of 6 ml/kg of predicted body weight (PBW), respiratory rate (RR) adjusted to ensure an arterial pH greater than 7.3 and an inspiration-to-expiration ratio of 1:1 to reach zero flow conditions at the end of each inspiration. The optimal level of positive end-expiratory pressure (PEEP) was set using Diagnostic View software (EIT Pulmovista 500, Dräger Medical Systems, USA). This software provides a visual representation of regional lung overdistention and collapse based on the pixel compliance loss at high PEEP (reflecting overdistention) or low PEEP (reflecting collapse) [13]. Briefly, PEEP was decreased stepwise from 16 cmH_2_O to 8 cmH_2_O in steps of 2 cmH_2_O every 2 min. Twenty consecutive breaths were analysed in the EIT at each PEEP step. The optimal PEEP level was determined as the crossing point of the overdistension and collapse curves during the decremental PEEP trial, to achieve a condition, where collapse and overdistension are jointly minimized [14]. The PEEP settings were maintained until the end of the protocol.

### Arterial blood gases

PaCO_2_, partial pressure of arterial oxygen (PaO_2_), and pH levels were analysed using arterial blood samples (GEM^®^ PREMIER™ 4000, Instrumentation Laboratory, Lexington, MA, USA).

### Respiratory mechanics

Respiratory signals of pressure, volume, flow, and RR were acquired and recorded continuously throughout the study using a proximal pneumotachograph and viewed on a Fluxmed monitor (MBMed, Buenos Aires, Argentina). Respiratory mechanics were assessed by performing 3-s inspiratory and expiratory holds under static and zero-flow conditions at the end of each step. The driving pressure was determined as the plateau pressure at the end of the inspiration cycle minus the total PEEP (PEEP_TOT_ = external PEEP + intrinsic PEEP) and respiratory system static compliance (C_RS_) as = V_T_/driving pressure. Minute ventilation (V̇E) was calculated using the following formula: V_T_ × RR. V_T_ was registered offline as an average of 20 breaths.

### Volumetric capnography

The expired CO_2_ was measured using a mainstream infrared sensor (Capnostat 5^®^; Respironics, OH, USA) and integrated into the Fluxmed monitor (MBMedCO_2_ Module) [15] (Additional file 1). The following estimators were registered:

### Area under the curve of the capnogram

- CO_2_ elimination per breath (V_T_CO_2_,_br_). The exhaled CO_2_ was obtained by integrating the flow and CO_2_ signals over the entire breath.
- CO_2_ output per minute (V̇CO_2_) is the product of V_T_CO_2_,_br_ × RR.
- Fraction of expired CO_2_ (F_E_CO_2_), calculated as the ratio between V_T_CO_2_,_br_, and expired V_T_. This represents the amount of CO_2_ diluted in each expired volume.
- The mean expired partial pressure of CO_2_ (P_E_CO_2_) was obtained from the fraction of expired CO_2_ multiplied by the barometric pressure minus the vapour pressure of water [16].

### Dead space and alveolar ventilation variables

- Bohr's dead space fraction (VD_Bohr_/V_T_): This ratio was obtained directly from the volumetric capnography by the following formula: VD_Bohr_/V_T_ = P_A_CO_2_–P_E_CO2/P_A_CO_2._ P_A_CO_2_ is the mean alveolar partial pressure of CO_2_ and was recorded at the midpoint of phase III of the capnogram [17].
- Airway dead space (VD_aw_) is the expired V_T_ portion that does not contain CO_2_. It was identified at the midpoint of the slope of phase II of the capnogram. VD_aw_ was normalized by the expired V_T_, resulting in the VD_aw_/V_T_ ratio.
- The alveolar dead space (VD_alv_) is the expired V_T_ portion that reaches the alveoli but does not participate in gas exchange. This is derived by subtracting VD_aw_ from VD_phys_. VD_alv_ is normalized by expired V_T_ and thus is represented as VD_alv_/V_T_ [18].
- Alveolar ventilation fraction (VT_alv_/V_T_), calculated as the ratio between alveolar ventilation and expired V_T_. Alveolar minute ventilation, was computed using the formula alveolar ventilation × RR indexed by PBW [19].

### Metrics related to global gas exchange

- Enghoff’s index was computed using the formula PaCO_2_–P_E_CO_2_/PaCO_2_. This index considers several variables influencing V̇/Q ˙, such as shunt effects and dead space [16].
- SIII refers to the steepness of the third phase of the capnogram curve. It is a sensible variable to quantify variations in V̇/Q ˙. In addition, it was normalized with F_E_CO_2_ to derive the SnIII for the corresponding expiratory cycle. Using SnIII allows a comparison of the slopes from breaths with different CO_2_ excretion rates, which could be expected to occur during V_T_ modifications [19].

All recorded signals were analysed offline using the mean value of the last 20 breaths. A more comprehensive explanation of the variables related to volumetric capnography is provided in Additional file 1.

Finally, the VR was calculated as ${\text{VR}}\, =  {(\,\mathop {\text{V}}\limits^{ \cdot } {\text{E}}_{{{\text{measured}}}} \left( {{\text{ml}}/{\text{min}}} \right)\, \times \,{\text{PaCO}}_{{2}} \left( {{\text{mmHg}}} \right))}\div{({\text{PBW}}\, \times \,{1}00\, \times \,{37}.{5 }\left( {{\text{PaCO}}_{{{\text{2predicted}}}} } \right))}$ [9].

### Electrical impedance tomography

A 16-electrode belt was placed in the fifth intercostal space, and continuous lung impedance was assessed using EIT (Pulmovista 500, Dräger Medical Systems, USA). Offline analysis of the EIT data was performed, and the following parameters were calculated:

- The global inhomogeneity index (GI) was computed by analysing a tidal image obtained from a 3-min recording. This involves summing the impedance changes for each pixel between the end of inspiration and expiration. Through this analysis, we assessed the dispersion of pixels over the median of the tidal image. To ensure consistent comparisons, we normalized this result based on the sum of impedance values for each pixel [20].
- Regional ventilation distribution was assessed using the impedance ratio (IR), computed by analysing a tidal image obtained from a 3-min recording. The impedance ratio (IR) divides the ventilation activity of the dorsal region by the ventral region ventilation activity of the functional EIT images [21]. IR > 1 represents a predominantly ventral region distribution ventilation, whereas IR < 1 represents a predominantly dorsal region ventilation distribution [22].

In the same 3-min EIT monitoring recording, twenty consecutive breaths from segments with minor variability in impedance were selected. From this subset of 20 breaths, the tidal variation of impedance (VTI) and end-expiratory lung impedance (EELI) were calculated by layers and quadrants.

- VTI represents the impedance change generated by the inspired gas during the respiratory cycle. It was obtained as the difference between the maximum and minimum point of impedance in a breathing cycle (VTI = TImax–TImin) [23].
- EELI corresponds to the impedance value at the end of expiration. Its changes have been correlated with changes in end-expiratory lung volume [24]. For further information regarding the variables analysed through EIT (Additional file 1).

### Positioning

After setting the baseline ventilatory strategy, we performed a 30-min stabilization period and then started the study protocol. Hill-Room Progressa™ beds were used to change the trunk inclination between different stages of the protocol.

### Study steps

We performed a sequential protocol that included three steps lasting 60 min each, during which trunk inclination was modified from 45° to 10° and then returned to 45°. The patient’s lower extremities were maintained without any inclination with respect to the floor surface.

**Fig. 1:**
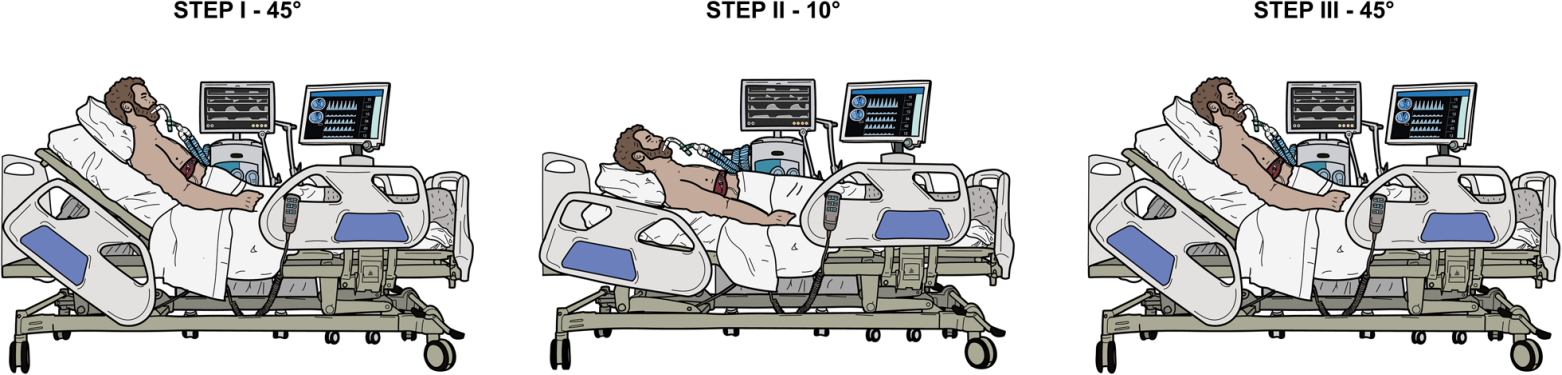
Study protocol. Graphic representation of the study. Each step was performed for 60 min. Mechanical ventilation, arterial blood gases, volumetric capnography and electric impedance tomography were recorded in each step

- STEP I: patients were positioned in a semirecumbent position at a 45° head-up inclination.
- STEP II: patients were positioned with the trunk at 10° in a supine-flat position.
- STEP III: patients were repositioned in the semirecumbent position at a 45° head-up inclination (Fig. 1).

At the end of each step, PaCO_2_, pH, and PaO_2_ over the fraction of inspired oxygen (PaO_2_/FiO_2_) were recorded. V_T_ and volumetric capnography were analysed offline using the mean value of the last 20 breaths of each step 2 min prior to the trunk inclination change. The hemodynamic variables and pulse oximetry results were continuously monitored (Multiparameter Spacelabs 91,393 Xprezzon^®^). The security procedure is included in Additional file 1.

### Primary outcomes

Our primary endpoint assessed the PaCO_2_ and VD_Bohr_/V_T_ reduction 60 min after a trunk inclination change to 10° with respect to the baseline conditions at an inclination of 45°.

### Statistical analysis

The sample size was calculated using a repeated-measures design [25]. We defined a clinically significant effect to be a mean reduction in PaCO_2_ of 5 mmHg with a standard deviation of ± 3 mmHg and a correlation using decay values of 0.5, power of 90%, and a probability of type I error of 0.01. Based on these data, the sample size was 22 participants [3]. The Shapiro‒Wilk test was performed to determine the distribution of continuous variables, while homoscedasticity was tested using the Levene or Bartlett test. According to their distribution, continuous variables are expressed as the mean and standard deviation (± SD) or median and interquartile range [IQR] and were analysed using ANOVA for repeated measures or the Friedman test, as appropriate. The Bonferroni correction and Dunn's post hoc test were used to compare the different study steps according to their distribution. A two-tailed *p* < 0.05 was considered statistically significant. Statistical analyses were conducted using RStudio version 4.3.1 (Integrated Development Environment, Boston, MA, USA).

## Results

Twenty-two patients with mild, moderate or severe ARDS completed the study and were included in the analysis. The baseline characteristics of the patients are shown in Table 1.

**Table 1:** Baseline characteristics of the patients included in the study

	*n* = 22
Baseline	Median [IQR]
Age (years)	58 [53–67]
APACHE II score	16 [12–19]
Body mass index (kg/m^2^)	30 [25–37]
Days of mechanical ventilation Prior to the study onset	4 [3–5]
Female/male	Number of patients
Female (*n*)	5
Male (*n*)	17
ARDS etiology	Number of patients
Bacterial pneumonia (*n*)	4
SARS CoV-2 pneumonia (*n*)	18
Use of vasopressors (*n*)	19
Baseline characteristics	Median [IQR]
Tidal volume (6 mL/kg/PBW)	351 [290–388]
Total PEEP (cmH_2_O)	10 [8–12]
Driving pressure (cmH_2_O)	12 [10–14]
Respiratory rate (bpm)	20 [18–24]
Minute ventilation (mL/kg/min)	120 [112–150]
C_RS_ (mL/cm/H_2_O)	33 [27–43]
PaO_2_/FiO_2_ (mmHg)	185 [151–210]
PaCO_2_ (mmHg)	43 [39–47]
pH (Arterial blood gases)	7.39 [7.36–7.42]
Electrical impedance tomography	
Impedance ratio (AU)	1.3 [0.8–1.8]
Inhomogeneity index (AU)	1.3 [1.1–1.5]

*APACHE II* Acute Physiology and Chronic Health disease Classification System II; *PEEP* positive end-expiratory pressure; *bpm* breaths/min; *PaO*_*2*_*/FiO*_*2*_ partial pressure of arterial oxygen over fraction of inspired oxygen; *PaCO*_*2*_ partial pressure of arterial CO_2_, *C*_*RS*_ compliance of the respiratory system; AU arbitrary units

Continuous data are expressed as medians [25th–75th percentiles, *IQR* interquartile range]

Categorical variables are expressed as absolute numbers

### Primary outcomes

**Fig. 2:**
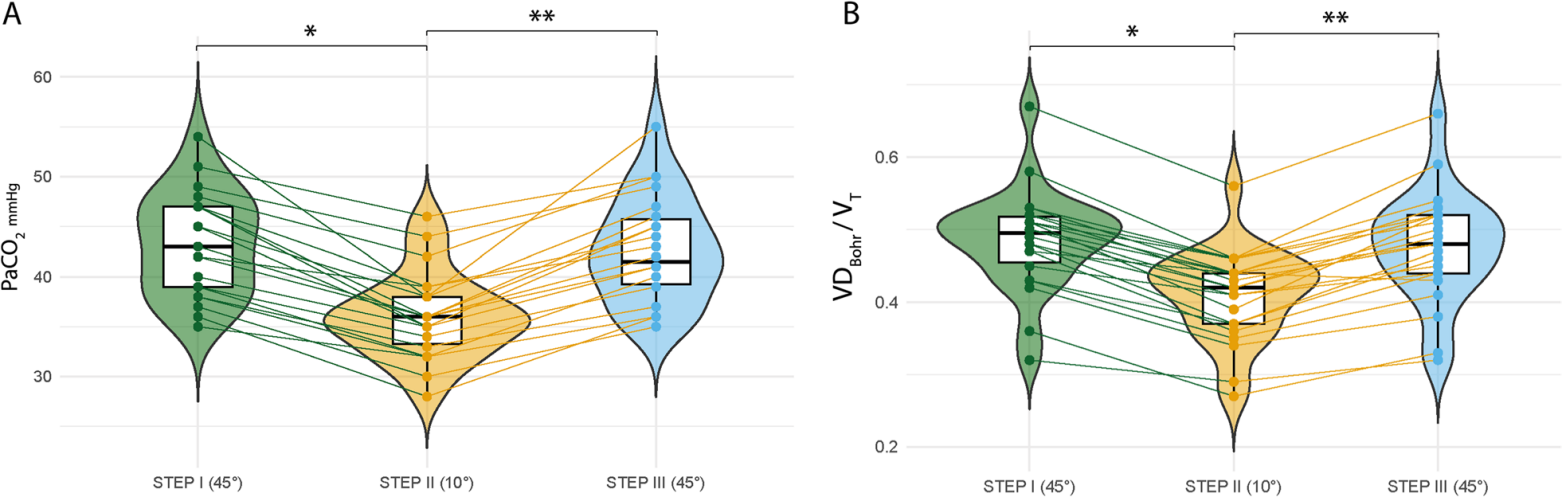
Effects of trunk postural change on PaCO2 and Bohr’s dead space. **A** PaCO_2_ and **B** Bohr’s dead space (VD_Bohr_/V_T_). Twenty-two ARDS patients on pressure-controlled ventilation underwent three 60-min steps in which the trunk inclination was changed from 45° (baseline) to 10° (intervention) and back to 45° (control). Scatter-box-violin plot summary values. The box depicts the 25th to 75th percentiles [IQR], the error bars denote the 10th to 90th percentiles, and the horizontal bar shows the median. Intergroup difference. Post hoc Bonferroni *P* values: *p < 0.05: STEP II (°10) vs. STEP I (45°); **p < 0.05: STEP III (45°) vs. STEP II (10°)

After changing the trunk inclination from a semirecumbent (45°) to a supine-flat (10°) position, PaCO_2_ decreased from 43 (± 5) to 36 (± 4) mmHg; *p* < 0.001), and VD_Bohr_/V_T_ decreased from 0.49 (± 0.07) to 0.41 (± 0.06); (*p* < 0.001) (Fig. 2A, B).

### Secondary outcomes

Mechanical ventilation: after changing trunk inclination from 45° to 10°, V_T_ increased from 371 (± 76) to 433 (± 84) mL *p* < 0.001), C_RS_ increased from 34 (± 10) to 41 (± 12) mL/cmH_2_O (*p* < 0.001) and alveolar minute ventilation rose from 120 [112–150] to 143 [128–168] mL/kg/min (*p* < 0.001) (Table 2).

**Table 2:** Effect of trunk inclination on ventilatory parameters, gas exchange, lung impedance, and hemodynamics

	STEP I—45° (*n* = 22)	STEP II—10° (*n* = 22)	STEP III—45° (*n* = 22)	*P* values
Respiratory mechanics				
Tidal volume (mL)	371 ± 76	433 ± 84 ^*a*^	365 ± 78^*b*^	*p* < 0.001
Minute ventilation (mL/kg/min)	120 [112–150]	143 [128–168]^*a*^	121 [109–149]^*b*^	*p* < 0.001
Driving pressure (cm/H_2_O)	12 [10–14]	12 [10–14]	12 [10–14]	–
PEEP (cm/H_2_O)	9 [7–11]	9 [7–11]	9 [7–11]	–
Total PEEP (cm/H_2_O)	10 [8–12]	10 [8–12]	10 [8–12]	–
Respiratory rate (bpm)	20 [18–24]	20 [18–24]	20 [18–24]	–
Ventilatory ratio (VR)	1.66 ± 0.39	1.43 ± 0.31	1.6 ± 0.39	*p* = 0.107
Arterial blood gases				
Arterial pH	7.39 ± 0,05	7.45 ± 0,05 ^*a*^	7.39 ± 0.06 ^*b*^	*p* = 0.003
PaCO_2_ (mmHg)	43 ± 5	36 ± 4 ^*a*^	42 ± 5 ^*b*^	*p* < 0.001
PaO_2_/FiO_2_ ratio (mmHg)	189 ± 33	196 ± 34	191 ± 28	*p* = 0.735
Volumetric capnography				
VD_Bohr_/V_T_	0.49 ± 0.07	0.41 ± 0.06^*a*^	0.48 ± 0.07^*b*^	*p* < 0.001
Enghoff's index of gas exchange	0.56 ± 0.08	0.48 ± 0.08^*a*^	0.54 ± 0.08	*p* = 0.006
V̇CO_2_ (ml/min)	191 ± 34	227 ± 38^*a*^	212 ± 44	*p* = 0.013
Alveolar ventilation (mL/kg/min)	74 ± 16	99 ± 21^*a*^	80 ± 17^*b*^	*p* < 0.001
VD_aw_/V_T_	0.36 ± 0.06	0.29 ± 0.06^*a*^	0.35 ± 0.06^*b*^	*p* = 0.002
VD_alv_/V_Talv_	0.19 ± 0.04	0.16 ± 0.03	0.18 ± 0.04	*p* = 0.066
VT_alv_/V_T_	0.51 [0.48–0.54]	0.58 [0.56–0.63]^*a*^	0.52[0.49–0.56]^*b*^	*p* < 0.001
SnIII (L^−1^)	1.45 [0.75–2.07]	0.58 [0.49–1.03]^*a*^	1.37 [0.73–1.69]^*b*^	*p* < 0.001
E_T_CO_2_ (mmHg)	40 ± 4	34 ± 4 ^*a*^	40 ± 5 ^*b*^	*p* < 0.001
Impedance electric tomography				
Impedance ratio (AU)	1.3 [0.8–1.8]	0.9 [0.5–1.3]^*a*^	1.36 [1.1–1.8]^b^	*p* < 0.001
Global Tidal variation (AU)	794 [444–1226]	841 [606–1333]	789 [442–1270]	*p* = 0.186
Global EELI (AU)	149 [90–367]	130 [91–256]	189 [90–319]	*p* = 0.421
Global Inhomogeneity index (AU)	1.3 [1.1–1.5]	1.2 [1.1–1,4]	1.15 [1.1–1.4]	*p* = 0.700
Basic hemodynamic				
Pulse pressure variation (%)	6 [5–7]	5.5 [5, 6]	6 [5–7]	*p* = 0.101
Heart rate (bpm)	72 ± 17	68 ± 16	69 ± 16	*p* = 0.353
Mean arterial pressure (mmHg)	76 ± 7	78 ± 8	77 ± 9	*p* = 0.133

*Bpm* breaths/min; *PaO*_*2*_*/FiO*_*2*_ partial pressure of arterial oxygen over fraction of inspired oxygen; *VD*_*alv*_*/VT*_*alv*_ alveolar dead space to alveolar expiratory tidal volume ratio, *VT*_*alv*_*/V*_*T*_ alveolar ventilation fraction; *SnIII* Capnogram at phase III normalized by the fraction of expired CO_2_; *E*_*T*_*CO*_*2*_ end-tidal partial pressure of CO_2_; *AU* arbitrary units

Values are presented as the mean ± standard deviation (SD) or median and interquartile range [IQR]

ANOVA for repeated measures and Friedman’s test were performed to compare multiple variables. Bonferroni correction and Dunn’s post hoc tests were used

^a^*p* < 0.05 s step (10°) vs. first step (45°)

^b^*p* < 0.05 third step (45°) vs. second step (10°)

^c^*p* < 0.05 third step (45°) vs. first step (45°). No patient showed significant differences between the first and third steps

**Fig. 3:**
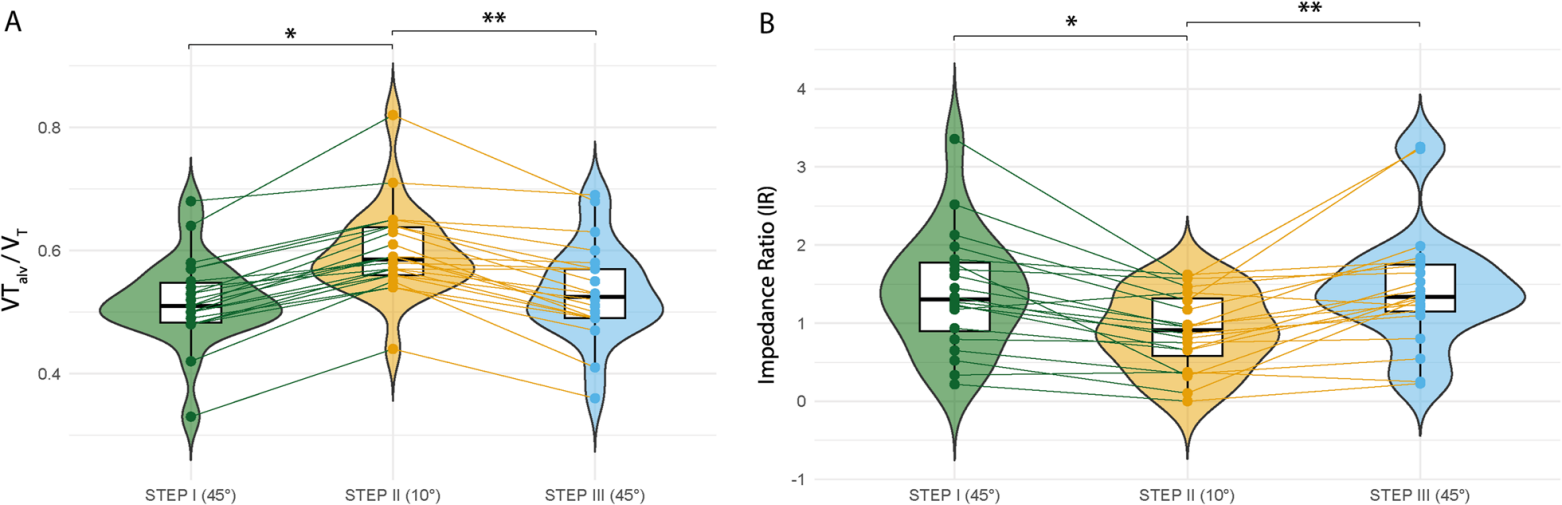
Effects of trunk postural change on the alveolar ventilation ratio (VT_alv_/V_T_) and impedance ratio (IR). **A** VT_alv_/V_T_ and **B** impedance ratio (IR). Scatter-box-violin plot summary values. The box depicts the 25th to 75th percentiles [IQR], the error bars denote the 10th to 90th percentiles, and the horizontal bar shows the median. Intergroup difference. Post hoc Bonferroni *P* values: *p < 0.05 step II (°10) vs. step I (45°); **p < 0.05 step III (45°) vs. step II (10°)

Gas exchange: after changing the trunk inclination from 45° to 10°, V̇CO_2_ increased from 191 (± 34) to 227 (± 38) mL/min (*p* < 0.001). Accordingly, an increase in VT_alv_/V_T_ from 0.51 [0.48 to 0.54] to 0.58 [0.56 to 0.63] (*p* < 0.001) was registered (Fig. 3A). SnIII dropped from 1.45 [0.75 to 2.07] to 0.58 [0.49 to 1.03]; *p* = 0.001) (Additional file 1: Fig. S3). The PaO_2_/FiO_2_ ratio did not significantly change among the different steps for trunk inclination adjustment (189 ± 33 vs. 196 ± 34; *p* = 0.735) (Table 2).

Electrical impedance tomography: in two patients, tracings were discarded due to poor-quality waveforms. After changing the trunk inclination from 45° to 10°, a decrease in the IR from 1.27 [0.83–1.78] to 0.86 [0.51–1.33] (*p* < 0.001) (Fig. 3B) was observed, indicating further air distribution from the ventral to dorsal lung regions. Regarding global EELI, no significant differences were observed with a change in trunk inclination (149 [90–367] vs. 130 [91–256]; *p* = 0.421). In the ventral region, there were no significant differences between the 45° and 10° trunk inclinations (102 [77–340] vs. 87 [15–136]; *p* = 0.360). The dorsal region did not yield significant differences (28 [11–59] vs. 8 [− 10 to 50]; *p* = 0.251).

**Fig. 4:**
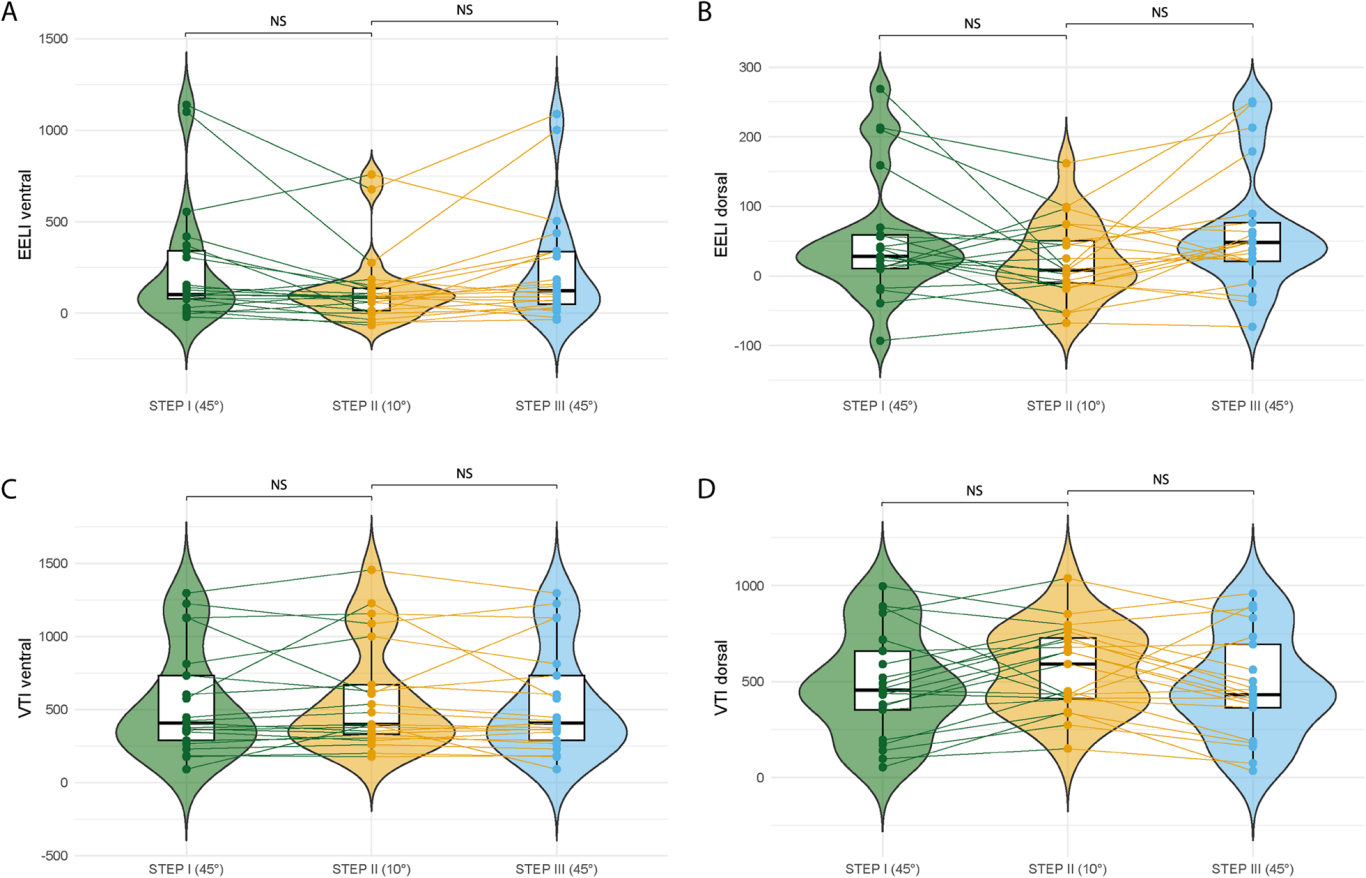
Effects of trunk postural change on regional end-expiratory lung impedance (EELI) and tidal variation of impedance (VTI). Ventral and dorsal EELI (**A** and **B**) and dorsal and ventral VTI (**C** and **D**). Scatter-box-violin plot summary values. The box depicts the 25th to 75th percentiles [IQR], the error bars denote the 10th to 90th percentiles, and the horizontal bar shows the median. Intergroup difference. Post hoc Bonferroni *P* values: *p < 0.05 step II (°10) vs. step I (45°); **p < 0.05 step III (45°) vs. step II (10°)

Global VTI changed from 794 [444–1226] to 841 [606–1333]; however, this difference was not statistically significant (*p* = 0.186). In the ventral region, there were no significant differences in the ventral VTI between 45° and 10° of trunk inclination (406 [287–732] vs. 398 [329–669], *p* = 0.932). The dorsal region revealed no difference between the two postural changes (456 [353–659] vs. 592 [412–727], *p* = 0.631) (Fig. 4). When VTI was analysed in four quadrants, the ventral regions of both the lungs and the dorsal right lung showed no significant changes. However, the dorsal left lung revealed an increase in VTI at 10° trunk inclination; *p* = 0.007 (Additional file 1: Fig. S6). In addition, there were no changes in the global homogeneity index (*p* = 0.700) across the different steps of the study. Finally, changes in V_T_, PaCO_2_, ventilatory variables, and variable measures in EIT were rapidly reversed once the patients were repositioned in the 45° semirecumbent position.

## Discussion

This clinical and physiological study investigated the effect of changing the trunk inclination from 45° to 10° in mechanically ventilated ARDS patients. The main findings revealed a decrease in PaCO_2_ levels and an improvement in ventilatory inefficiency. Furthermore, we observed a significant shift in ventilation distribution from ventral to dorsal lung regions. These observations reinforce the physiological and clinical impact of supine-flat trunk positioning on the respiratory mechanics of patients with ARDS. In addition, our results provide new insights into the direct influence of this positioning on ventilatory distribution and the balance between ventilatory efficiency and inefficiency.

### Change in trunk inclination and its effects on respiratory system mechanics

Studies have consistently indicated that a shift from a semirecumbent to a supine-flat position in patients with ARDS receiving passive ventilation significantly affects respiratory mechanics. In a study on 20 C-ARDS patients, Marrazzo et al. found that changing the trunk inclination from a semirecumbent (40°) to supine-flat (0°) position decreased driving and transpulmonary pressure and increased both chest wall and lung compliance [3]. Similarly, Mezidi et al. observed that patients with “classical” ARDS connected to mechanical ventilation who underwent a postural change from a semirecumbent head-up position at 30° to a supine-flat 0° position presented a significant reduction in lung and chest wall elastance after 10 min of observation [1]. We did not use esophageal pressure monitoring to measure lung and chest wall compliance, but similar to previous experiences, we found that trunk inclination toward the supine-flat position at 10° increased the C_RS_ while maintaining constant values of driving pressure.

### Effects of trunk inclination adjustment on ventilation distribution

In general, the transition of mechanically ventilated patients from a semirecumbent to a flat-supine position leads to decreased airway pressure by reducing intra-abdominal pressure and improving chest compliance [2, 26]. These physiological adjustments provide a plausible explanation for the main result of our study obtained by EIT: a decrease in the impedance ratio (IR). This decline in IR implies an enhanced ventilation distribution in the dorsal lung regions. In turn, most patients demonstrate improved C_RS_ and alveolar ventilation in tandem with this effect. These findings suggest that the supine-flat position allows the lungs to operate within a more favorable range of their pressure‒volume curves, reducing the strain and stress on the lungs [27]. On the other hand, although we did not directly measure the end-expiratory lung volume (EELV), we utilized EIT to assess the EELI, allowing us to evaluate regional ventilation distribution. Interestingly, we did not observe any significant differences in the global and regional EELI between the two positions (45° vs. 10°), as shown in Fig. 4. This finding aligns with the heterogeneous effects reported in previous studies investigating EELV [2], which exhibited variations among individual patients. Consequently, these data do not support the hypothesis that changes in EELI explain the observed positive effects on respiratory mechanics, as suggested in a previous study [28]. Therefore, it is not feasible to attribute the enhancements in respiratory mechanics, increased CO_2_ exhalation, and improved ventilation distribution in the dorsal regions to a possible increase in the recruitment of dorsal areas during the transition from a semirecumbent to a supine position.

In Step II, although there was an increase in VT, this was not reflected in an increase in VTI when analysed by lung layers (ventral and dorsal lung areas). Approximately 70% (14/20) of the participants experienced increased global and regional VTI when trunk inclination was adjusted from 45° to 10°, in contrast to 100% of the patients who presented an increase in VT. Previous studies have demonstrated a good correlation between these variables [29]. However, Mosing et al. performed a study to explore the relationship between the progressive rise of VT from 4 to 20 ml/kg and VTI. They observed only a good correlation when VT was 8–20 mL/kg^−1^ [30]. We believe that the low VT of 6 ml/kg^−1^ we set and the change in this volume generated with a trunk inclination is likely insufficient to discriminate the difference.

While the changes observed in IR indicated further air distribution from the ventral to the dorsal lung regions, this shift was not reflected in a higher dorsal VTI when analysed by layers. Therefore, it becomes apparent that the variations in VTI are less robust than those observed in IR. This discrepancy is likely due to the enhanced ability of IR to accurately capture changes in the ratio between the ventral and dorsal regions. However, when we performed quadrant-based analyses of the right and left lungs, only the left dorsal region showed a marked increase in VTI at 10° of trunk inclination. This was the only significant finding that showed concordance with the increase in VT and the decrease in IR, where the latter expressed a more significant impedance activity in the lung dorsal areas with changes in trunk inclination from 45 to 10°. This finding suggests that factors such as regional lung compliance or the superimposed pressures on the lung tissue in this specific region could be affected differently during the position change from semirecumbent to supine-flat position.

### Effects of trunk inclination adjustment on gas exchange

Few studies have evaluated the effects of changes in chest inclination on ventilatory efficiency/inefficiency at different degrees of inclination. Available data show that when patients move from a semirecumbent position to a supine-flat position, it can generate PaCO_2_ and VR reduction, but only in specific study populations.

In our study, minute ventilation was increased to expense further V_T_ when the patients were placed in the 10° supine-flat position. These effects were accompanied by improvements in V̇CO_2_ and VD_Bohr_/V_T_, which suggested better alveolar ventilation without further overdistension. This is supported by the observation of a marked drop in SnIII when patients were placed in the supine-flat (10°) position, implying effective CO_2_ exchange [31]. SnIII has demonstrated a good correlation with the gold standard (*MIGET*) for measuring V̇/Q ˙ globally in injured lungs [32]. Although a SnIII decrease could also be interpreted as more efficient lung perfusion and lower shunt, it is impossible to determine, with our study design, whether a change in trunk inclination to a supine-flat position could improve this circulatory inefficiency.

Marrazzo et al. also observed a significant decrease in PaCO_2_, but the effect was lower than that in our trial [3]. These differences could be due to several factors. First, we used pressure-controlled ventilation, which increased the minute ventilation when changing the patient's trunk inclination. In contrast, Marrazzo et al. maintained constant-minute ventilation throughout their study. Second, Marrazzo's study's evaluation time was shorter than ours (15 min vs. 60 min). Despite the possible influence of evaluation times on these outcomes [33], we did not observe any significant differences in the parameters recorded by volumetric capnography when we compared the effects in the supine-flat position at 15- and 60-min intervals, as detailed in the Additional file 1: Table S1). Therefore, the findings derived from volumetric capnography reveal that significant changes in CO_2_ clearance occur swiftly after a change in trunk inclination, and these changes sustain themselves with minimal fluctuations throughout the 60-min evaluation.

In contrast, Dellamonica et al. did not find significant variability in PaCO_2_ levels with changes in thoracic inclination in a cohort of 40 ARDS patients [2]. We cannot rule out the influence of the etiology of ARDS (COVID-19 and typical ARDS) on these results.

When a change in trunk inclination is generated toward a semirecumbent position, the abdominal contents can push up against the diaphragm, reducing the amount of space available for the lungs to expand, which could affect lung volume and CO_2_ exhalation [7, 34]. Studies have reported progressive increases in intra-abdominal pressure in patients placed in a semirecumbent position, and others have shown a correlation between intra-abdominal pressure and impairment in lung function [38]. However, there is limited clinical evidence that directly links changes in trunk inclination to airway pressure and gas exchange impairment [1]. In addition, it is unclear how changes in trunk inclination interact with global and regional lung perfusion, regional transpulmonary pressure, and superimposed lung tissue in dependent and nondependent regions. Therefore, these physiological conditions need to be explored.

In our study, no basic hemodynamic changes were observed at 180 min of measurement between the different steps of the study. In addition, no side effects were registered in the short term, and the procedure was completed in 22 patients.

This study boasts some significant strengths, including its alignment with earlier research and the rapid reversal of effects when patients are returned to a 45-degree position. In essence, these attributes not only reinforce the validity of our research but also provide a compelling affirmation of previous findings [1–3]. Finally, it is also important to note that the degree of inclination of the bed should be not only recorded in clinical practice but also required in research studies that perform physiological assessments of patients with respiratory failure, because, as we have seen, the results can vary significantly depending on the angle of inclination of the bed, which can generate erroneous conclusions from the results obtained.

## Limitations

First, the slender design of the EIT electrode belt was confined to a lung segment located in the fifth intercostal space. Although it can extend the imaging area of the lung by up to 10 cm from this plane, this conventional placement likely does not consistently capture data from regions farther from the belt [39]. This limitation may vary from person to person, depending on factors, such as the size, amount of subcutaneous tissue, and extent of lung collapse. Second, volumetric capnography can only yield results on global ventilatory function, limiting its ability to determine the regional (dorsal/ventral or apical/juxtadiaphragmatic) effects of the intervention. Third, the potential impact of hemodynamics on CO_2_ exhalation is difficult to exclude without advanced hemodynamic monitoring. Fourth, we did not measure intra-abdominal pressure to compare different positions or establish an association with physiological effects on the lungs. Finally, the number of patients included in the study was relatively small.

## Conclusions

A change in trunk inclination from a semirecumbent (45 degrees) to a supine-flat position (10 degrees) improved VD_Bohr_/V_T_ and reduced PaCO_2_ in ARDS patients. This effect is associated with increased V_T_ and C_RS_ and further favourable impedance ventilation distribution toward the dorsal lung regions. This study highlights the importance of considering trunk inclination as a modifiable determinant of physiological parameters. The angle of trunk inclination is essential information that must be reported in ARDS patients.

### Supplementary Information

**Additional file 1.** The TIDieR reporting guidelines. Methods. Volumetric capnography. Electrical impedance tomography. Security procedure. **Figure S1.** Enghoff`s index gas exchange. **Figure S2.** Airway dead space. **Figure S3.** The slope of phase III (SIII). **Table S1.** Volumetric capnography. **Table S2.** Tidal variation of impedance. **Figure S4.** Ventral and dorsal VTI. **Table S3.** End-Expiratory lung impedance. **Figure S5.** Ventral and dorsal EELI. **Table S4.** Quadrant-based right and left lung analyses using. **Figure S6.** A Dorsal region of the right lung. **B** Dorsal region of the left lung. **Table S5.** Global Inhomogeneity index (GI) analysis.

## Data Availability

The data sets used and analysed during the current study are available from the corresponding author upon reasonable request.

## Abbreviations

PaCO_2_: Partial pressure of carbon dioxide
VR: Ventilatory ratio
ARDS: Acute respiratory distress syndrome
EELV: End-expiratory lung volume
$V$/Q ˙: Ventilation/perfusion ratio
VD_Bohr_/V_T_: Bohr’s dead space
EIT: Electrical impedance tomography
V_T_: Tidal volume
PBW: Predicted body weight
RR: Respiratory rate
PEEP: Positive end-expiratory pressure
PaO_2_: Partial pressure of arterial oxygen
C_RS_: Respiratory system compliance
$$V$$E: Minute ventilation
V_T_CO_2_,_br_: CO_2_ elimination per breath
$V$CO_2_: CO2 output per minute
F_E_CO_2_: Fraction of expired CO_2_
P_E_CO2: Mean expired partial pressure of CO_2_
VD_phys_: Physiological dead space
P_A_CO_2_: Mean alveolar partial pressure of CO_2_
SIII: The slope of Phase III
SnIII: FECO2-normalized SIII
VD_aw_: Airway dead space
VD_alv_: Alveolar dead space
VT_alv_/V_T_: Alveolar ventilation fraction
GI: Global inhomogeneity index
IR: Impedance ratio
VTI: Tidal variation of impedance
EELI: End-expiratory lung impedance
PaO_2_/FiO_2_: Partial pressure of oxygen to the fraction of inspired oxygen
