# Supplementary material for: Effects of changes in trunk inclination on ventilatory efficiency in ARDS patients: quasi-experimental study
Source: Intensive Care Med Exp. 2023 Sep 27;11:65. doi: 10.1186/s40635-023-00550-2 (PMC10533449; doi:10.1186/s40635-023-00550-2)
Supplement: Supplementary file 1 — Additional file 1. The TIDieR reporting guidelines. Methods. Volumetric capnography. Electrical impedance tomography. Security procedure. Figure S1. Enghoff`s index gas exchange. Figure S2. Airway dead space. Figure S3. The slope of phase III (SIII). Table S1. Volumetric capnography. Table S2. Tidal variation of impedance. Figure S4. Ventral and dorsal VTI. Table S3. End-Expiratory lung impedance. Figure S5. Ventral and dorsal EELI. Table S4. Quadrant-based right and left lung analyses using. Figure S6. A Dorsal region of the right lung. B Dorsal region of the left lung. Table S5. Global Inhomogeneity index (GI) analysis. [file 40635_2023_550_MOESM1_ESM.pdf]

## SUPPLEMENTARY DIGITAL FILES

# TITLE

# Effects of trunk inclination change on carbon dioxide removal efficiency in ARDS patients: Quasi-experimental study

Martín H. Benites; David Torres; Fabian Poblete; Francisco Labbe; María C. Bachmann; Tomas E. Regueira; Leonardo Soto; Andrés Ferre; Jorge Dreyse; and Jaime Retamal

**Table of Contents**

The TIDieR reporting guidelines …………………………………………………………………. Page 3

Methods …………………………………………………………………………………….. Page 6

Volumetric capnography ………………………………………………………… Page 6

Electrical impedance tomography ………………………………………………. Page 8

Security procedure ……………………………………………………………... Page 10

eFigure 1. Enghoff`s index gas exchange ……………………………………………… Page 12

eFigure 2. Airway dead space ……………………………………………………...………. Page 13

eFigure 3. The slope of phase III (SIII) …….……………..…………………………...... Page 14

eTable 1. Volumetric capnography ……… ..……………………………...………..…… Page 15

eTable 2 Tidal variation of impedance ……………………………..……………. Page 16

eFigure 4. Ventral & dorsal VTI …………………………………………………….………….. Page 16

eTable 3. End-Expiratory lung impedance ……………………………………….……….. Page 17

eFigure 5. Ventral & dorsal EELI …………………………………………………….………. Page 17

eTable 4. Quadrant-based right and left lung analyses using …………………………… Page 18

eFigure 6A Dorsal region of the right lung ……………………………………………… Page 18

eFigure 6B Dorsal region of the left lung ……………………………………………… Page 18

eTable 5. Global Inhomogeneity index (GI) analysis …………………………………… Page 19

- **The TIDieR reporting guidelines (Template for Intervention Description and Replication) Checklist:**

Information to include when describing an intervention and the location of the information. https://www.equator-network.org/reporting-guidelines/tidier/

| | |
|---|---|
| **1- BRIEF NAME** | |
| Provide the name or a phrase that describes the intervention. | Page 9,10 |
| **2- WHY** | |
| Describe any rationale, theory, or goal of the elements essential to the intervention. | Page 5 |
| **3- WHAT** | |
| Materials: Describe any physical or informational materials used in the intervention, including those provided to participants or used in intervention delivery or in the training of intervention providers. Provide information on where the materials can be accessed (e.g. online appendix, URL). | Page 7,8,9<br>** |
| Procedures: Describe each of the procedures, activities, and/or processes used in the intervention, including any enabling or support activities. | ** |
| **4- WHO PROVIDED** | |
| For each category of intervention provider (e.g. psychologist, nursing assistant), describe their expertise, background and any specific training given. | ** |
| **5- HOW** | |
| Describe the modes of delivery (e.g. face-to-face or by some other mechanism, such as internet or telephone) of the intervention and whether it was provided individually or in a group. | Page 8,9 |
| **6- WHERE** | |
| Describe the type(s) of location(s) where the intervention occurred, including any necessary infrastructure or relevant features. | Page 5,8<br>** |
| **7- WHEN and HOW MUCH** | |

| | |
|---|---|
| Describe the number of times the intervention was delivered and over what period of time including the number of sessions, their schedule, and their duration, intensity or dose. | Page 8,9 |
| **8- TAILORING** | |
| If the intervention was planned to be personalized, titrated or adapted, then describe what, why, when, and how. | * N/A |
| **9- MODIFICATIONS** | |
| If the intervention was modified during the course of the study, describe the changes (what, why, when, and how). | * N/A |
| **10- HOW WELL** | |
| Planned: If intervention adherence or fidelity was assessed, describe how and by whom, and if any strategies were used to maintain or improve fidelity, describe them. | Page 7, 8, 9,10 |
| Actual: If intervention adherence or fidelity was assessed, describe the extent to which the intervention was delivered as planned. | *N/A |

**Authors -** use N/A if an item does not apply to the described intervention.

** Detailed information of some ítems of The TIDieT Checklist

3.- WHAT

Materials:

Describe any physical or informational materials used in the intervention, including those provided to participants or used in intervention delivery or the training of intervention providers. Provide information on where the materials can be accessed (e.g. online appendix, URL).

- $Pa_{CO2}$ measurements were performed at the end of each phase using a blood gas analyzer (GEM® PREMIER™ 4000). https://www.werfen.com/benelux/en/gem-premier-4000

- The Fluxmed monitor (MBMed, Buenos Aires, Argentina) was used to measure and record the expired CO2. This device, equipped with a specialized MBMed CO2 Module, integrates dedicated software to analyse ventilatory variables in real-time automatically. https://mbmed.com/co2-module/

- The PulmoVista® 500 device (Dräger Medical GmbH, Lübeck, Germany) was used to monitor lung impedance. https://www.draeger.com/Products/Content/pulmovista-500-pi-9066581-es-es.pdf

- Respiratory signals of pressure, volume, flow, respiratory rate (RR), and inspiratory/expiratory time were acquired and recorded continuously throughout the study by a proximal pneumotachograph and viewed on the Fluxmed monitor (MBMED, Buenos Aires, Argentina). The mechanical ventilator used was Drager Infinity C500 (Germany). https://www.draeger.com/Products/Content/evita-v500-sw2-pi-9072800-en-1510-2.pdf.

- Hill-Room ProgresaTM beds were used to assess the changes in trunk inclination. These beds have an electronic monitor that provides information regarding the inclination angle of the selected bed. The bed angle was digitally selected. https://www.hillrom.com/en/products/progress-bed-system/

- A multiparametric monitor (Spacelabs 91393 Xprezzon®) continuously monitored blood pressure, heart rate, pulse pressure, pulse pressure, and temperature. https://www.spacelabshealthcare.com/products/patient-monitoring-connectivity/patient-monitoring/xprezzon/

4.- For each category of intervention provider (e.g. psychologist, nursing assistant), describe their expertise, background, and any specific training given.

Martín Benites and respiratory therapists Fabián Poblete and Francisco Labbe executed the study's measurements and data recording. As the lead researcher, Martín Benites brought his expertise as a medical doctor specializing in Critical Care and held a master's degree in epidemiology. He is pursuing his Doctorate in Medical Sciences at the Pontificia Universidad Católica de Chile. It should be noted that all the researchers involved are specialists in Critical Care.

6.- Describe the type(s) of location(s) where the intervention occurred, including any necessary infrastructure or relevant features.

The study was conducted in Clínica las Condes, Santiago, Chile. Clínica las Condes. The ICU has sixteen beds with individual rooms, receiving highly complex patients from the whole country.

## Methods

### Volumetric capnography

The Fluxmed monitor (MBMed, Buenos Aires, Argentina) was employed in this study, featuring a mainstream infrared sensor (Capnostat 5®; Respironics, OH, USA) capable of measuring expired CO2 (with a range of 0-150 mm Hg, accuracy ± 2 mmHg, and response time of less than 60 ms). This sensor was integrated with the Fluxmed monitor via an MBMed CO2 module. The device incorporates MATLAB ®-programmed software (Mathworks, Natick, MA, USA) to analyse ventilation in volumetric capnograms, utilizing a mathematical algorithm (Levenberg–Marquardt) that correlates tidal volume with exhaled CO2. Consequently, it was feasible to derive data on dead space and alveolar ventilation. Data were recorded using Fluxview® software for analysis. The recorded signals were analysed offline using the mean value of the last 20 breaths of each step."

Volumetric capnography variables:

- $V_TCO_{2,br}$: Amount of $CO_2$ removed per exhaled volume, represented as the area under the capnogram curve. This variable is very sensitive to changes in pressure, volume, temperature, and cardiac output. Thus, a continuous registry of all these variables was carried out to legitimize the updated results.

- Fraction of $CO_2$ expiration ($F_ECO_2$): the relationship between $CO_2$ and exhaled $V_T$. (2). It is the amount of CO2 diluted in each expired volume.

- Mean alveolar partial pressure of $CO_2$ ($P_ACO_2$): this value was recorded at the midpoint of phase III of the capnogram using specially designed and validated software (2).

- Mixed-expired partial pressure of $CO_2$ ($P_ECO_2$): PECO2 was obtained directly from the volumetric capnography by the following formula: $P_ECO_2 = (V_TCO_{2,br} /VTe) * $ (barometric pressure – vapour pressure of water) (3).

The barometric pressure was fixed, and the vapour pressure of water was calculated according to the body temperature of each patient. Volumes were automatically compensated for body temperature and water vapour pressure.

- Bohr's dead space ratio ($VD_{Bohr}/V_T$): This ratio was obtained directly from the volumetric capnography by the following formula: $VD_{Bohr}/V_T= P_ACO_2 – P_ECO_2/P_ACO_2$ (3).

- Enghoff´s index. Due to the involvement of different mechanisms of mismatching ventilation/perfusion, it is denominated as an index of gas exchange. For its calculation, it was necessary to obtain arterial blood gases. It was obtained using the following formula: Enghoff index gas exchange = $PaCO_2 – P_ECO_2/PaCO_2$ (2).

- Airway dead space ratio ($VD_{aw}/V_T$): The relationship between the airway dead space ($VD_{aw}$) and expired $V_T$. The midpoint of phase II represents the boundary between the airway dead space ($VD_{aw}$) and alveolar ventilation ($VT_{alv}$) according to Fowler's concept (4). The volume exhaled up to this point corresponds to anatomical dead space. When the exhaled volume exceeds the midpoint of Phase II, alveolar ventilation occurs. The midpoint of Phase II was calculated using the device software.

- Bohr’s dead space or physiological dead space ($VD_{phys}$): The sum of the $VD_{aw}$ + alveolar dead space ($VD_{alv}$) (2). Additionally, it can be calculated using the following mathematical formula: $(P_ACO_2 – P_ECO_2/P_ACO_2) \times V_T$

All of these variables were calculated directly by volumetric capnography. The mean value of 20 breaths in the last minute of each *step* was obtained for each variable studied.

Example: Capnogram of one breath. Comparison between steps I (Figure A) and II (Figure B).

Step I. Trunk inclination at 45°

B- Step II. Trunk inclination at 10°

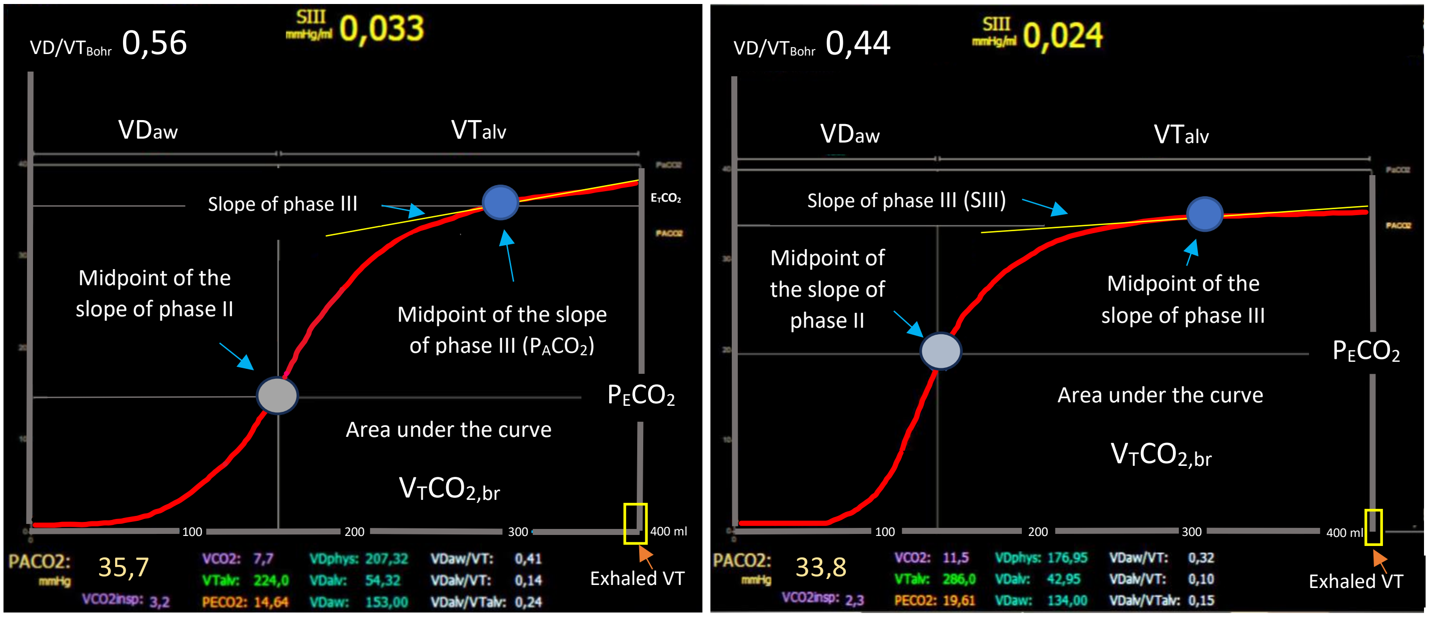

**Electrical impedance tomography**

A 16-electrode belt was placed in the fifth intercostal space, and continuous lung impedance was assessed using EIT (Pulmovista 500, Dräger Medical Systems, USA). Offline analysis of the EIT data was performed, and the following parameters were calculated:

a. **Global inhomogeneity index (GI):** The GI was computed by analysing a tidal image obtained from a 3-minute recording. This involves summing the impedance changes for each individual pixel in the period between end-inspiration and end-expiration. Through this analysis, we assessed the dispersion of pixels over the median of the tidal image. To ensure consistent comparisons, we normalized this result based on the sum of impedance values for each pixel. A value close to 0 indicates low dispersion compared to the median, indicating a more homogeneous distribution of ventilation among the pixels. Conversely, as the value deviated from 0, the dispersion of the pixels relative to the median increased, indicating a more heterogeneous ventilation pattern.

$$GI = \sum_{x,y \in \text{lung}} \left| DI_{xy} - \text{median}\left(DI_{\text{lung}}\right) \right| / \sum_{x,y \in \text{lung}} DI_{xy}.$$

DI denotes the value of the differential impedance in the TV images; DIxy is the pixel in the identified lung area; DIlung are all pixels in the lung area under observation.

Image obtained from our previous study. *Bachmann MC,* et al. *(2018) Electrical impedance tomography in acute respiratory distress syndrome. Crit Care 22:263*

***b.*** **Impedance ratio (IR):** The impedance ratio divides the ventilation activity of the ventral region by the dorsal ventilation activity of the tidal images and may indicate both lung recruitment and derecruitment. It was determined by analysing a 32 by 32-pixel tidal image derived from a 3-minute recording. This image, represented by numerical values in a spreadsheet, allowed us to calculate the ratio between the ventral segment tidal image and the dorsal segment tidal image of impedance. The calculation involved summing the numerical values of the upper ROI of 16 x 32 ventral pixels and dividing it by the sum of the 16 x 32 dorsal pixels.

The impedance ratio (IR) was used to divide the ventilation activity into the upper (ventral) region and dorsal ventilation. This IR index has proven to be a sensitive parameter for tracking vertical changes in ventilation, which can be induced by processes such as recruitment, derecruitment, or overdistension. (7)

IR > 1 represents a predominantly ventral distribution, whereas IR < 1 represents a predominantly dorsal distribution. Briefly, the ventral region corresponded to regions of interest (ROI) 1 and 2, and the dorsal region corresponded to ROIs 3 and 4. (8)

An example of a tidal image, represented by the numerical values in a spreadsheet, is provided below.

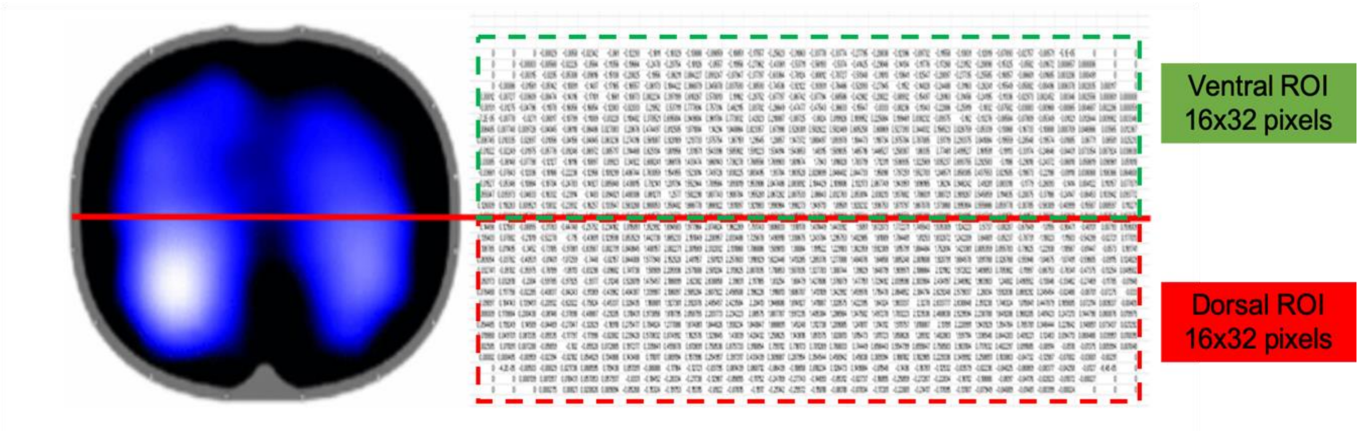


In the same 3-minute EIT monitoring recording, 20 consecutive breaths from segments with minor variability in impedance were selected. From this subset of 20 breaths, we calculated the tidal variation of impedance (VTI) and end-expiratory lung impedance (EELI). We will now provide a detailed explanation of these calculations.

- **a.** VTI represents the impedance change generated by the inspired gas during the respiratory cycle. It was obtained as the difference between the maximum and minimum point of impedance in a breathing cycle (TVI= TImax - TImin)
- **b.** EELI corresponds to the impedance value at the end of expiration. Its changes have been correlated with changes in end-expiratory lung volume.

Example of EIT tracing.

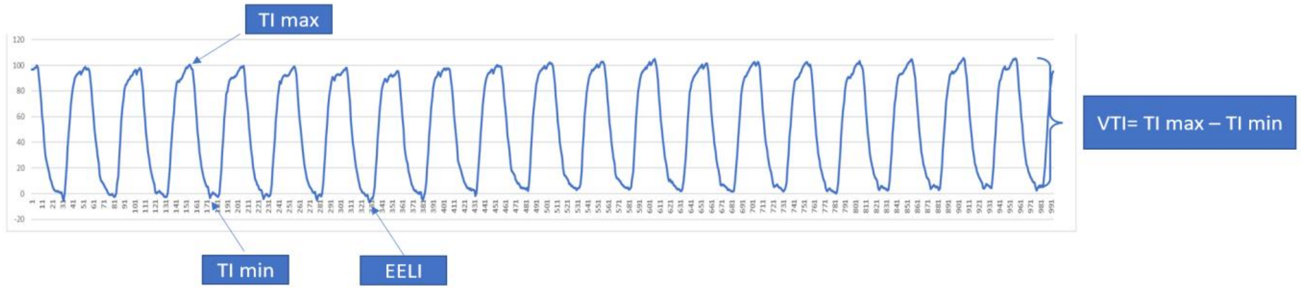


Tracing of 20 consecutive breaths was obtained from a 3-minute recording at the end of the protocol phase. Each curve represents the impedance change of breath. The highest point on each impedance curve represents the Tidal Impedance Max (TI max), whereas the lowest point represents the Tidal Impedance Min (TI min). The difference between TI max and TI min for each breath provides each breath's VTI. The TI min of each curve represents the End-Expiratory Lung Impedance (EELI) value for that breath. Both VTI and EELI values were derived from an average of 20 breaths.

**Security procedure**

Adverse clinical events were predefined to interrupt the study protocol and to ensure patient safety. These criteria included a drop of more than 10% in baseline pulse oximetry, a decrease in mean arterial pressure exceeding 20% from baseline, a fall below 65 mmHg, or a heart rate increase of more than 20% from baseline. Additionally, if the dose of vasopressors was increased by more than 50% for any reason, it was also considered a criterion for study suspension. It is important to note that therapeutic interventions for each patient were maintained throughout the evaluation period, ensuring the continuity of appropriate care and treatment.

**Supplemental Digital Content References**


1. Tusman G, Scandurra A, Böhm SH, Suarez-Sipmann F, et al. Model fitting of volumetric capnograms improves calculations of airway dead space and slope of phase III. *J Clin Monit Comput* 2009; 23:197-206.
2. Suerez-Sipmann F, Bohm SH, Tusman G. Volumetric capnography: the time has come. *Curr Opin Crit Care*, 2014; 20:333-9
3. Tusman G, Sipmann FS, Borges JB, et al. Validation of Bohr dead space measured by volumetric capnography. *Intensive Care Med*, 2011; 37:870–874

4. Fletcher R, Jonson B (1981) The concept of deadspace with special reference to the single breath test for carbon dioxide. Br J Anaesth 53:77–88.
5. Bachmann MC, Morais C, Bugedo G, Bruhn A, Morales A, Borges JB, Costa E, Retamal J (2018) Electrical impedance tomography in acute respiratory distress syndrome. Crit Care 22:263
6. Yang, L., Dai, M., Möller, K., Frerichs, I., Adler, A., Fu, F., & Zhao, Z. (2021). Lung regions identified with CT improve the value of global inhomogeneity index measured with electrical impedance tomography. Quantitative imaging in medicine and surgery, 11(4), 1209–1219.
7. Kunst, P. W., Vazquez de Anda, G., Böhm, S. H., Faes, T. J., Lachmann, B., Postmus, P. E., & de Vries, P. M. (2000). Monitoring of recruitment and derecruitment by electrical impedance tomography in a model of acute lung injury. Critical care medicine, 28(12), 3891–3895.
8. Bachmann, M. C., Cruces, P., Díaz, F., Oviedo, V., Goich, M., Fuenzalida, J., Damiani, L. F., Basoalto, R., Jalil, Y., Carpio, D., Hamidi Vadeghani, N., Cornejo, R., Rovegno, M., Bugedo, G., Bruhn, A., & Retamal, J. (2022). Spontaneous breathing promotes lung injury in an experimental model of alveolar collapse. Scientific reports, 12(1), 12648.

**Supplementary digital eFigure 1.**

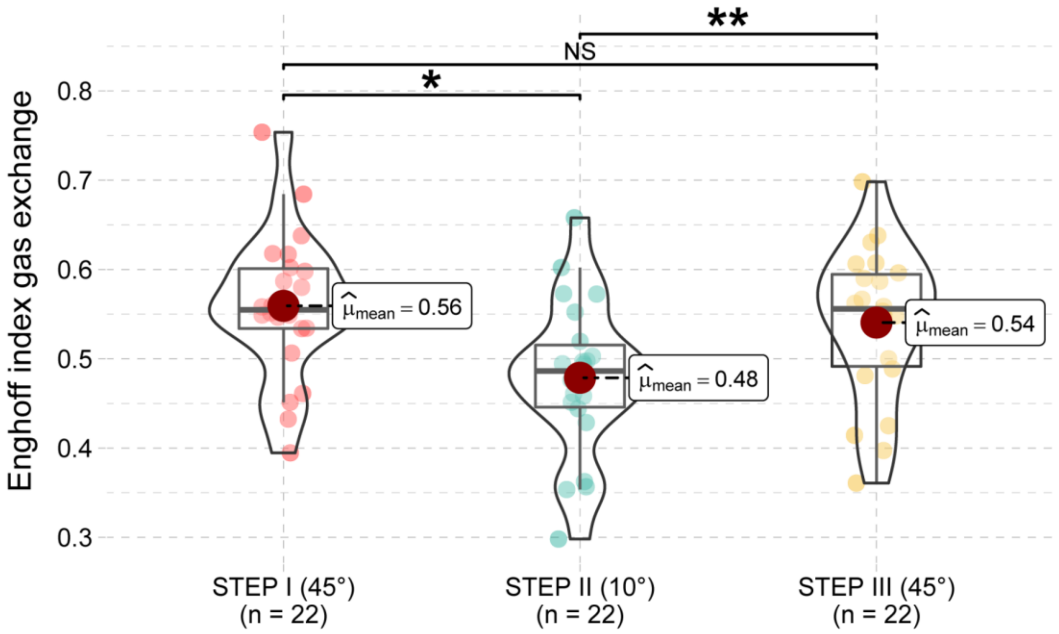


eFigure 1- Enghoff`s index gas exchange. Twenty-two ARDS patients on pressure-controlled ventilation underwent three 60-minute steps in which trunk inclination was changed from 45° (baseline) to 10° (intervention) and back to 45° (control). Significant reduction from SETP I to STEP II and rapid reversal when patients were placed on 45° again (STEP III). Scatter-box-violin plot summary values. The box depicts the 25th to 75th percentiles [IQR], the error bars denote the 10th to 90th percentiles, and the horizontal bar shows the median. Additionally, it is possible to observe the dot distribution of each patient and the mean value (Bordeaux colour). Intergroup difference. Post hoc Bonferroni P values: *P < 0.05-STEP II (°10) versus STEP I (45°); **P < 0.05 STEP III (45°) versus STEP II (10°).

**Supplementary digital eFigure 2.**

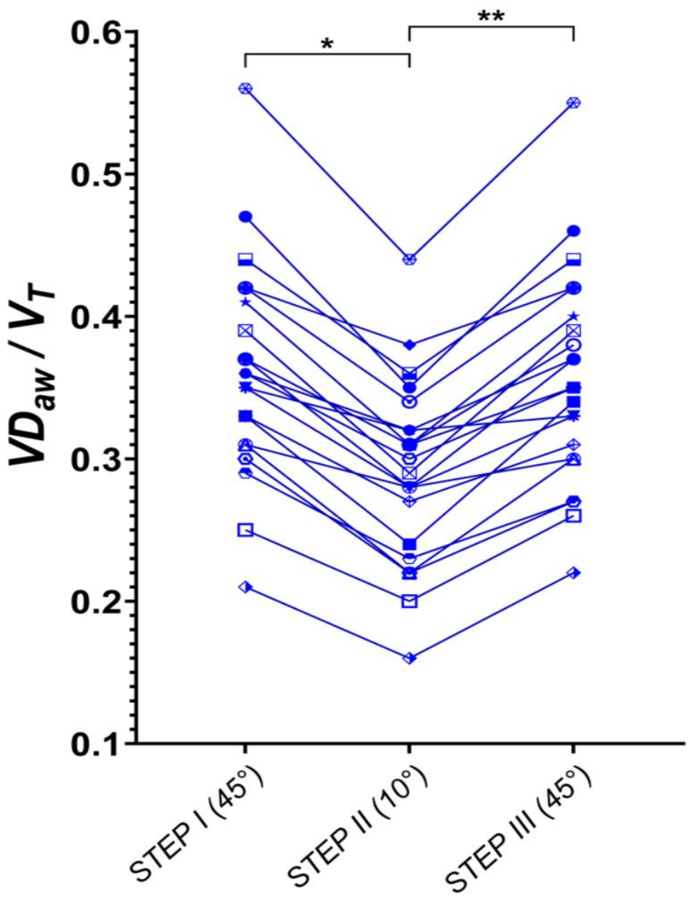


eFigure 2- Airway dead space ($VD_{aw}/V_T$). Different trunk inclinations are represented in each step. Each patient was assigned a symbol and kept constant in all graphs to allow their individual evaluation. Post hoc Dunn Test P values: *p=< 0.05-second step (°10) versus first step (45°); **p=< 0.05 third step (45°) versus second step (10°). GraphPad Prism version 9.3.1 (GraphPad Software, San Diego, CA, USA)

**Supplementary digital eFigure 3.**

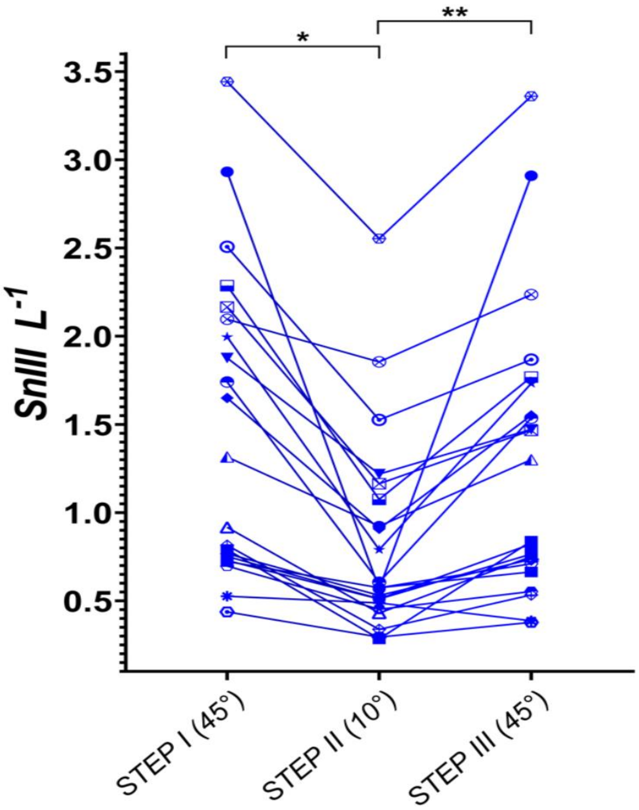


eFigure 3. The slope of phase III (SIII) of the capnogram normalized by the Fraction Expired of $CO_2$ (SnIII). Different trunk inclinations are represented in each step. Each patient was assigned a symbol and kept constant in all graphs to allow their individual evaluation. Post hoc Dunn Test P values: *p=< 0.05-second step (°10) versus first step (45°); **p=< 0.05 third step (45°) versus second step (10°). GraphPad Prism version 9.3.1 (GraphPad Software, San Diego, CA, USA)

**eTable1.** Comparative analysis of results at 15 and 60 minutes in Step II

| | STEP II | | *p* - values |
|---|---|---|---|
| | **10° 15 min** | **60 min** | |
| **Volumetric Capnography** | | | |
| $\dot{V}CO_2$ (ml/min) | 225 ± 40 | 227 ± 38 | $p = 0.883$ |
| $VD_{Bohr}/V_T$ | 0.42 ± 0.05 | 0.41 ± 0.06 | $p = 0.073$ |
| Alveolar ventilation (mL/kg$_{-PBW}$/min) | 98 ± 20 | 99 ± 21 | $p = 0.904$ |
| $E_TCO_2$ mmHg | 35 ± 3 | 34 ± 4 | $p = 0.538$ |

$\dot{V}CO2$ is the CO2 output per minute, $VD_{Bohr}/VT$ Bohr´s dead space ratio, $E_TCO_2$ End-tidal $CO_2$

Results of volumetric capnography 15 and 60 min after the patients were positioned at a 10° angle inclination position. No significant differences were observed between the two study periods (15 min vs. 60 min) in variables that express CO2 exhalation.

**eTable 2. Tidal variation of impedance (VTI)**

| | STEP I 45° Median (IQR) | STEP II 10° Median (IQR) | STEP III 45° Median (IQR) | p-value |
|---|---|---|---|---|
| VTI ventral (AU) | 406 [287–732] | 398 [329–669] | 428 [257–762] | 0.932 |
| VTI dorsal (AU) | 456 [353–659] | 592 [412–72] | 432 [364–694] | 0.631 |

**eFigure 4. Ventral & dorsal VTI**

The percentage change from STEP I to STEP II was calculated using the following equation:

Percentage of Change = ((VTI value 10° - VTI value 45°) / VTI value 45°) × 100

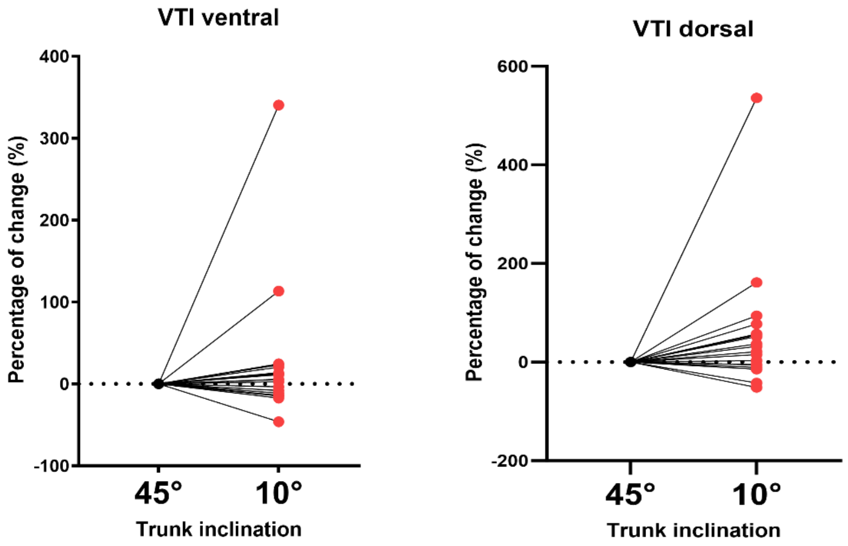


The basal conditions were assigned a value of zero for all patients. From this baseline value, the percentage changes in VTI were plotted regionally, corresponding to changes in the chest inclination from 45° to 10°.

No significant differences were observed in the percentage changes resulting from adjusting trunk inclination from 45° to 10°.

**eTable 3. End-expiratory lung impedance (EELI)**

| | STEP I 45° Median (IQR) | STEP II 10° Median (IQR) | STEP III 45° Median (IQR) | p-value |
|---|---|---|---|---|
| Ventral EELI (AU) | 102 (77–340) | 87 (15–136) | 122 [48 - 336] | 0.360 |
| Dorsal EELI (AU) | 28 (11–59) | 8 (-10 - 50) | 48 [19 - 76] | 0.251 |

**eFigure 5. Ventral & dorsal EELI**

The percentage change from STEP I to STEP II was calculated using the following equation:

Percentage of Change = ((EELI value 10° - EELI value 45°) / EELI value 45°) × 100

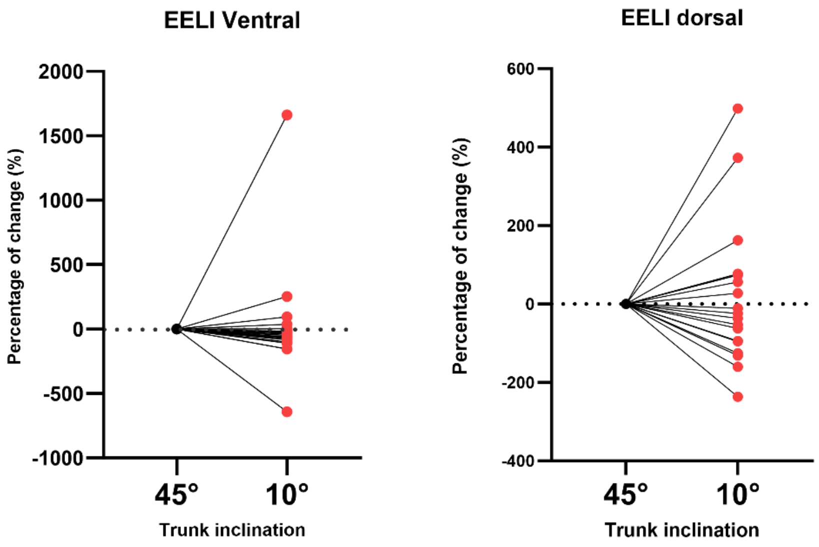


The basal conditions were assigned a value of zero for all patients. From this baseline value, the percentage changes in EELI were plotted regionally, corresponding to changes in the chest inclination from 45° to 10°. No significant differences were observed in the percentage changes resulting from adjusting trunk inclination from 45° to 10°.

**eTable 4. Quadrant-based right and left lung analyses using electrical impedance tomography.**

| | STEP I | STEP II | STEP III | p -value |
|---|---|---|---|---|
| VTI ventral right lung (AU) | 194 [ 78 - 285] | 190 [59 - 304] | 201 [72 - 320] | p = 0.752 |
| VTI ventral left lung (AU) | 171 [79 - 284] | 140 [101 - 316] | 149 [68 - 277] | p = 0.212 |
| VTI dorsal right lung (AU) | 182 [95 -252] | 211 [133 - 297] | 180 [106 - 226] | p= 0.430 |
| VTI dorsal left lung (AU) | 225 [120 - 288] | 298 [211 - 403]* | 212 [119 - 295]** | p= 0.007 |
| | | | | |
| EELI ventral right lung (AU) | 26 [5 - 67] | 15 [- 7 - 29] | 29 [2 - 49] | p= 0.522 |
| EELI ventral left lung (AU) | 60 [34 - 160] | 50 [14 - 131] | 100 [40 - 210] | p= 0.449 |
| EELI dorsal right lung (AU) | 1 [ -11 - 15] | - 2 (-14 - 14) | 4 [-15 - 13] | p= 0.626 |
| EELI dorsal left lung (AU) | 25 [8 - 52] | 27 [3 - 43] | 21 [6 - 37] | p= 0.349 |

* p < 0.05 second step (10°) versus first step (45°). ** p < 0.05 third step (45°) versus second step (10°).

VTI demonstrated notable interpatient variability. When adjusting for trunk inclination, the ventral regions of both the lungs and the dorsal right lung showed no significant statistical changes. In contrast, the dorsal left lung revealed a marked increase in VTI at a 10° trunk inclination. This last finding is in concordance with the reduction observed in IR, where both variables highlighted a better ventilation distribution in the dorsal regions of the lung.

**eFigure 6A Dorsal region of the right lung**

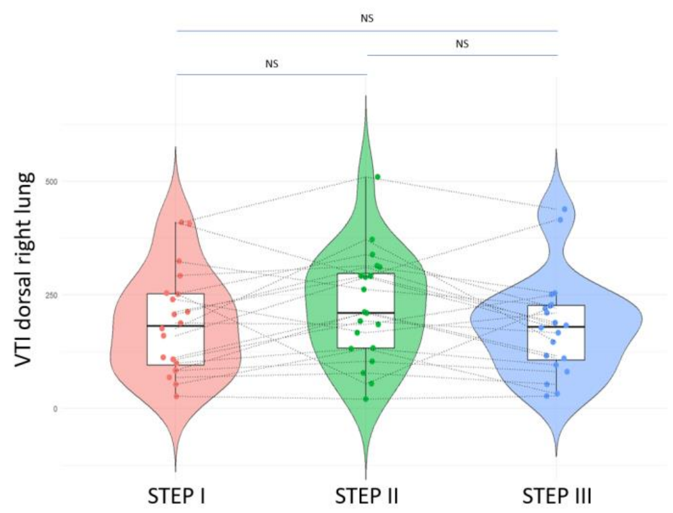


**eFigure 6B Dorsal region of the left lung**

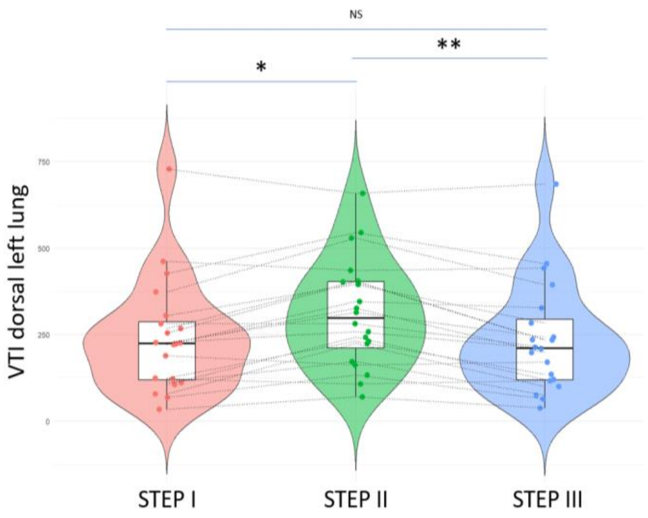


* p < 0.05 second step (10°) versus first step (45°). ** p < 0.05 third step (45°) versus second step (10°).

EELI also displayed marked inter-patient variability, yet no statistically significant differences were discerned across any of the lung quadrants under examination.

**Global Inhomogeneity index (GI) analysis**

**eTable 5.** Global Inhomogeneity index (GI) analysis. The values were obtained using two distinct calculation methods.

| | STEP I | STEP II | STEP III | p -value |
|---|---|---|---|---|
| GI pixel selection (ventilated lung area) (AU) | 1.56 [1.3 – 2.6] | 1.42 [0.95 – 2.1] | 1.62 [1.2 – 2.5] | $p = 0.732$ |
| GI (lung area in the GI calculation) (AU) | 1.3 [1.1 - 1.5] | 1.2 [1.1 – 1.4] | 1.15 [1.1 – 1.4] | $p = 0.700$ |

Adjusting the trunk tilt to 10° did not induce any statistically significant change in GI. In our analysis of 20 patients focused only on ventilated lung areas, nine showed values lower than those calculated using GI, with ventilated and non-ventilated lungs.

Interestingly, the mean GI values calculated using only selected pixels of ventilated areas were higher than those derived from calculations without such a specific pixel area selection (GI calculated with ventilated and non-ventilated lung areas).

Despite disparities in analytical approaches, the trend in GI with the two calculation forms remained consistently aligned across the three stages of the study. As this is a repeated-measures study evaluating the change in GI with different trunk angles, the change factor is probably more relevant than the absolute value according to different calculation methods.
